# Letter to the Editor: Effect of Social Activity on All‐Cause Dementia Among South Korean Elders: A Retrospective Cohort Study

**DOI:** 10.1002/hsr2.71302

**Published:** 2025-09-23

**Authors:** Mahnoor Umrani, Aiman Faiz, Muhammad Zain Ul Haq

**Affiliations:** ^1^ Isra University Hyderabad Pakistan; ^2^ Karachi Metropolitan University Karachi Pakistan; ^3^ Department of Medicine Dow University of Health Sciences Karachi Pakistan

Dear Editor,

We read with great interest the article published by Jeong et al. [1]. This looks into how social interaction and dementia incidence relate to elderly Korean seniors. Although the author's topic is pertinent, we would like to politely bring up a few methodological and interpretive issues that need more debate.

First, the authors stress the structural and cognitive components of social contact (such as group activities); they don't examine the established psychological processes that could be responsible for the observed correlation. According to the stress‐buffering theory, psychological anguish is lessened by stress when people think that they have social support [2]. This indirect protective effect through emotional well‐being is worth discussing, as longitudinal studies have shown that social isolation and poor social relationships significantly increase the risk of dementia [3]. A more thorough explanation of how social involvement may affect cognitive outcomes over time would be possible by including such psychosocial elements.

Second, the changing character of older individuals' social participation may not be adequately captured by the author's concept of “social activity.” Their study excludes digital forms of social connection like email, video calling, and online group involvement, but it includes conventional, in‐person types of activity like volunteer groups and religious gatherings. Given mounting evidence that internet use may help older persons' cognitive and emotional well‐being, especially for those who face social, geographic, or physical obstacles to face‐to‐face interaction, this is a noteworthy omission. Randomized trials have shown that daily internet‐based conversational engagement significantly improves executive functioning and memory in older adults, suggesting a neuroprotective effect [4]. Since both chronic loneliness and depression are established risk factors for cognitive decline and dementia, these digital interactions may offer neuroprotective effects by enhancing mental health and promoting cognitive stimulation. By leaving out these kinds of participation, the study may understate the extent of protective social activity and, as a result, how social connections could lower the prevalence of dementia. Lastly, however, the fact that the authors have taken demographics, physical activity, and socioeconomic background into consideration is amazing, but I would like to draw attention to a significant methodological flaw that could significantly affect how the results are interpreted. Personality traits and baseline cognitive reserves were not taken into consideration. The two most significant determinants of cognitive health among the five‐factor model traits are neuroticism and conscientiousness. Higher levels of neuroticism and lower levels of conscientiousness have been linked to a higher probability of Alzheimer's disease and dementia in general. Additionally, some new research indicates that a higher risk of incident dementia is associated with lower levels of extraversion, openness, and agreeableness [5]. So, the observed correlation between social engagement and dementia risk may be exaggerated or misattributed if these important individual difference variables are left out, making it challenging to accurately evaluate the independent neuroprotective benefit of social participation.

We appreciate the author's thoughtful efforts and hope that our comments will benefit the ongoing discussion.

## Author Contributions

**Mahnoor Umrani:** conceptualization, writing – original draft. **Aiman Faiz:** writing – review and editing. **Muhammad Zain Ul Haq:** supervision, final approval.

## Ethics Statement

The authors have nothing to report.

## Conflicts of Interest

The authors declare no conflicts of interest.

## Transparency Statement

The lead/Corresponding author (Aiman Faiz) affirms that this manuscript is an honest, accurate, and transparent account of the study being reported; that no important aspects of the study have been omitted; and that any discrepancies from the study as planned (and, if relevant, registered) have been explained.

## Data Availability

Data sharing is not applicable to this article as no datasets were generated or analysed during the current study.
